# Global nitrogen deposition inputs to cropland at national scale from 1961 to 2020

**DOI:** 10.1038/s41597-023-02385-8

**Published:** 2023-07-26

**Authors:** Srishti Vishwakarma, Xin Zhang, Achim Dobermann, Patrick Heffer, Feng Zhou

**Affiliations:** 1 University of Maryland Center for Environmental Science Appalachian Laboratory, Frostburg, MD USA; 2 Currently located at Joint Global Change Research Institute, Pacific Northwest National Laboratory, College Park, MD USA; 3 International Fertilizer Association, Paris, France; 4 Institute of Carbon Neutrality, Laboratory for Earth Surface Processes, College of Urban and Environmental Sciences, Peking University, Beijing, China

**Keywords:** Element cycles, Agriculture

## Abstract

Nitrogen (N) deposition is a significant nutrient input to cropland and consequently important for the evaluation of N budgets and N use efficiency (NUE) at different scales and over time. However, the spatiotemporal coverage of N deposition measurements is limited globally, whereas modeled N deposition values carry uncertainties. Here, we reviewed existing methods and related data sources for quantifying N deposition inputs to crop production on a national scale. We utilized different data sources to estimate N deposition input to crop production at national scale and compared our estimates with 14 N budget datasets, as well as measured N deposition data from observation networks in 9 countries. We created four datasets of N deposition inputs on cropland during 1961–2020 for 236 countries. These products showed good agreement for the majority of countries and can be used in the modeling and assessment of NUE at national and global scales. One of the datasets is recommended for general use in regional to global N budget and NUE estimates.

## Background & Summary

**Fig. 1:**
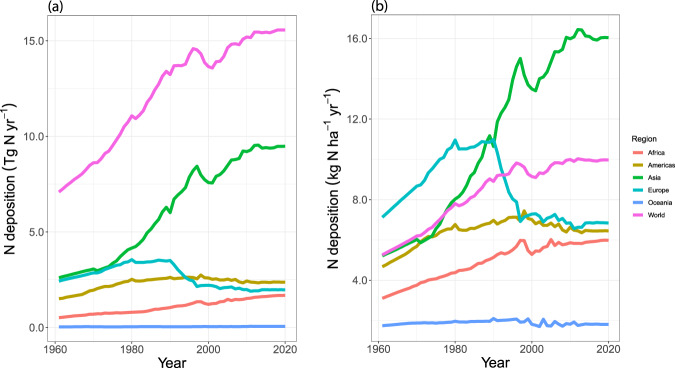
Historical trend of cropland N deposition in (**a**) Tg N yr^−1^ and (**b**) kg N ha^−1^ yr^−1^ by regions. Data are adopted from FAOSTAT’s Cropland Nutrient Budget^26^.

Atmospheric deposition of reactive nitrogen (N) is one of the important N inputs to cropland^1^ and, in many world regions, has been increasing over the last few years due to growing agricultural and industrial activities^2^ (Fig. 1). While the increasing trends of N deposition have slowed down, or even been reversed in some countries such as the USA^3^, many countries still show signs of rising N deposition. For instance, India’s average N deposition increased from about 13 kg N ha^−1^ yr^−1^ to 18 kg N ha^−1^ yr^−1^ between the 1980s and 2000s^4,5^. High rates of N deposition not only cause aggravating environmental and public health concerns, but also influence the N balance and N use efficiency (NUE) on cropland and other agricultural land^6^. Consequently, quantifying the N deposition to cropland is an important component for evaluating the efficiency and the fate of N in crop production. Assessment of N budgets on a national scale enables countries to become increasingly committed to improving NUE through new technologies, better practices, and policy measures^7,8^.

Generally, N deposition can be directly measured as wet deposition and dry deposition, with the sum of both providing estimates of the total N deposition (i.e., both wet and dry). Among these, wet deposition is most readily quantified with available precipitation chemistry data, while dry deposition is challenging to quantify by monitoring networks because it is sparsely distributed than wet deposition, affecting the estimate of dry fluxes. The uncertainty with dry deposition is high due to large spatiotemporal variations of gases, particles, and weather conditions. Hence, to date, only a limited number of regional monitoring sites around the world (e.g., Deposition of Biogeochemically Important Trace Species (DEBITS)/Africa), and the Clean Air Status and Trends Network (CASTNET)) have acquired the capability to measure long-term dry deposition^9^. Given these challenges and disparities in regional measurements of N deposition rate, a large number of studies have been utilizing an ensemble of global atmospheric chemistry-transport models to calculate N deposition at the global scale, usually expressed as total deposition (wet and dry)^10^. These global scale products have been used in multiple studies to assess N deposition inputs and N budgets under different land uses^11–15^.

Nitrogen deposition rates to cropland have been used for assessing the efficiency of N use and potential N loss on national scales in many studies^11,12,16,17^. Typically, in such N budgets, the N deposition inputs are calculated by overlaying the modelled N deposition maps with a cropland distribution map, as well as country boundaries (Table 1). However, the spatial and temporal coverages of these maps vary among studies, and the precision of the maps varies too. Based on a summary of the methods, data availability, and survey of expert opinions, we chose to use two different global N deposition maps (i.e., Atmospheric Chemistry and Climate Model Intercomparison Project (ACCMIP)^10^ and Wang *et al*.^18–20^) and two cropland maps (i.e., Land-Use Harmonization 2 (LUH2)^21^ and History of the Global Environment database (HYDE)^22^) in this study (Tables 1 and 2). While some region-specific models that emphasize cropland^23–25^ might yield a more precise evaluation of N deposition, their restricted spatial extent doesn’t align with the objectives of this study, which seeks to quantify N deposition for the majority of countries worldwide. In addition, few regional models provide a time span that extends over the extensive period from 1961 to 2020. Combining these different sources of maps resulted in four products of N deposition inputs on cropland at annual scale for 236 countries for the period 1961–2020. We compare our N deposition estimates with existing literature and ground observations, and discuss the four products and their usability. The four products developed were the results of the combinations of four maps: **AH**: ACCMIP + HYDE, **AL**: ACCMIP + LUH2, **WH**: Wang *et al*. + HYDE, and **WL**: Wang *et al*. + LUH2. On balance, we recommend using the WL data product for global estimates of N deposition on cropland. This data product was included in the Cropland Nutrient Budget database, a joint release by the Food and Agriculture Organization of the United Nations (FAO), the International Fertilizer Association (IFA), and various research groups^26^, available at https://www.fao.org/faostat/en/#data/ESB.

**Table 1:** A summary of data sources used for quantifying N deposition in global datasets.

Datasets	References	Data Sources and Methods by Variable	
		N Deposition	Land area
Zhang 2015; Zhang Reorganized^†^	Zhang *et al*.^13^	Used deposition map from Bouwman *et al*.^12^ and cropland distribution map of Ramankutty *et al*.^57^	Harvested area from FAOSTAT
Lassaletta	Lassaletta *et al*.^39,40^	Dentener *et al*.^30^, Bouwman *et al*.^58^	Used a combination of harvested area and cropland from FAOSTAT
Conant and Dorich	Conant *et al*.^16^	Dentener *et al*.^30^	Cropland area from FAOSTAT
Bodirsky with (or without) forage^‡^	Bodirsky *et al*.^11,17^	ACCMIP^10^, LUH2^21^	Cropland area from LUH2^21^, based on Klein Goldewijk *et al*.^59^
IMAGE	Bouwman *et al*.^12^	Deposition map from Dentener *et al*.^30^ for year 2000. Using historical and projected emission, the deposition maps are scaled to other years.	Land cover data from HYDE 3.1^59^
Gerber and Mueller	Mueller *et al*.^45,46^, West *et al*.^47^	Deposition map for 2000 from Dentener *et al*.^30^	Cropland area: FAOSTAT, irrigated area: Portmann *et al*.^60^, crop area: Monfreda *et al*.^61^, pasture area: Ramankutty *et al*.^57^
Chang *et al*.	Skalský *et al*.^48^, Herrero *et al*.^49^, Valin *et al*.^50^, Havlík *et al*.^51^, Chang *et al*.^52^	Atmospheric N deposition for the year 2000 is derived from the International Global Atmospheric Chemistry (IGAC)/Stratospheric Processes and Their Role in Climate (SPARC) Chemistry-Climate Model Initiative (CCMI) N deposition fields^62^.	Harvested area from crop-specific and spatially explicit SPAM dataset is harmonized with cropland land cover from GLC2000^48^. Further, at regional level to harvested area from FAOSTAT. Some additional adjustment of total cropland to total arable land from FAOSTAT.
FAO	FAOSTAT Soil N Budget Domain^44^	Zhang *et al*.^13^	Cropland area from FAOSTAT
Wang *et al*.	Wang *et al*.^18^, Shang *et al*.^19^, Wang *et al*.^20^	Dry and wet N depositions for 1850–2100 simulated by the global aerosol chemistry climate model LMDZ-OR-INCA	Land cover data from HYDE 3.1^59^
Lu and Tian	Lu and Tian^41^, Zhang *et al*.^42^	Not Available	Cropland area from HYDE 3.2^22^
Nishina with (or without) double cropping^*^	Nishina *et al*.^43^	Not Available	Cropland area from the Harmonized Global Land Use map (LUHa) v1.0^63^.

^†^Both datasets were used by Zhang *et al*., using the same data processing steps, but with input data belong to different years from FAO. Zhang 2015 downloaded data from 2014, while Zhang Reorganized used data from 2018, respectively.

^*^Two datasets were used by Nishina. Double cropping region is based on global crop use intensity (CUI) map developed by Siebert *et al*.^64^. “Without double cropping” indicate excluding the N fertilizer input in double cropping region from the inputs.

^‡^Two datasets were used by Bodirsky, one dataset include forage crop, while other exclude it in the budget.

**Table 2:** Datasets used for the analysis.

Datasets	Spatial resolution	Spatial coverage	Temporal resolution	Temporal coverage	Type of N deposition quantified/modeled
N deposition maps					
ACCMIP^10^	0.5° × 0.5° (55.5 × 55.5 km)	Global	Annual	1850–2100	Dry and wet deposition of NHy and Nox
Wang *et al*.^18–20^	1.25° × 2.5° (~138 × 277.5 km)	Global	Annual	1850, 1960, 1970, 1980, 1990, 1997–2013	Total N deposition
Cropland maps					
LUH2^21^	0.25° × 0.25° (~27.7 × 27.7 km)	Global	Annual	850–2100	
HYDE3.2^22^	5′ × 5′ (0.0833 × 0.08333 ~9 × 9 km)	Global	Annual	1960, 1970, 1980, 1990, 2000–2017	
Station networks					
UK ECN^65^	4 stations	Country	Daily	1992–2015	Wet deposition of NHy, NOx and total N
USA NADP^66^	357 stations	Country	Annual	1978–2019	Wet deposition of NHy, NOx and total N
China^67^	268 stations	Country	Annual	1980–2018	Bulk N deposition (annual NH4–N plus NO3–N input from precipitation)
EANET^37^ East Asia	7 stations	Country	Annual	2000–2020	Wet and dry bulk N deposition
N emission maps					
EDGAR^27^	0.1° × 0.1° (~11.1 × 11.1 km)	Global	Annual	1970–2018	Total emissions of NOx and NH3

Note: ACCMIP: Atmospheric Chemistry and Climate Model Intercomparison Project; LUH2: Land-Use Harmonization 2; HYDE: History of the Global Environment database; UK: United Kingdom; ECN: Environmental Change Network; USA: United States of America; NADP: National Atmospheric Deposition Program; EANET: Acid Deposition Monitoring Network in East Asia; EDGAR: Emissions Database for Global Atmospheric Research.

## Methods

### Data sources

The datasets used for developing N deposition at a national scale include N deposition maps, and cropland maps. Both types of maps have different spatiotemporal resolutions (Table 2). The N deposition maps from ACCMIP include dry and wet deposition of NHy and NOx, while Wang *et al*. comprise bulk N deposition without discriminating between the types of N deposition. Cropland maps (i.e., LUH2 and HYDE) with finer resolution were adjusted by summing their values to a coarser resolution to match the grid resolution of the N deposition map. Since the N deposition data from Wang *et al*. are available for a shorter time period than the period targeted in this study, we used N emission data from the Emissions Database for Global Atmospheric Research (EDGAR)^27^ to extrapolate and interpolate the values of N deposition to complete the time series from 1961–2020. We compared our national scale estimates with observed N deposition data from measurement station networks in the UK, China, the USA, and East Asia, and with the N deposition estimates used in 14 N global budget datasets from Zhang *et al*.^14^.

The ACCMIP dataset is a multi-model ensemble dataset providing the mean of N deposition across 11 models^10^. Out of 11 models, 10 models include NOx chemistry, while only 5 models include NHy chemistry. All the models varied in their spatial resolution to model deposition (see supplementary information in Lamarque *et al*.^10^). Both dry and wet depositions of oxidized and reduced N are estimated and simulated for five time slices: 1850, 1980, 2000, 2030, and 2100 years. The deposition estimates are averaged across models that were originally at monthly time scale. The emissions used for modeling deposition include anthropogenic (including shipping and aircraft), biomass burning, and natural emissions (such as soil NOx). Natural emissions were not harmonized across models. The models are calibrated for the 1980–2000 period and represent climate change in increments of 10 years rather than a specific meteorological year. Only the model estimates of wet deposition are evaluated against measurement stations in North America, Europe, and Asia with no information on the number of sites. This comparison showed ACCMIP results lower in North America and Europe, and worse in Asia compared with previous studies^10^.

In contrast to ACCMIP, Wang *et al*. data, which also have dry and wet N depositions for the period of 1850–2100, were quantified by LMDZ-OR-INCA (Laboratoire de Météorologie Dynamique -OR- INteraction with Chemistry and Aerosols) models. This is the global aerosol chemistry climate model, which couples the General Circulation Model and Aerosol Model^28,29^. In the model, emissions data are from oceans (NH3), vegetation emissions (NO), and other agricultural activities (such as fertilizer application), and fuel combustion (NOx and NHy). To capture N in aerosols and gases and simulate global dry and wet deposition, the LMDZ-INCA was run at a spatial resolution of 1.27° latitude by 2.5° longitude for the years 1850, 1960, 1970, 1980, 1990, and 1997–2013. The detailed methodology to model transport and removal processes is in Wang *et al*.^20^. Model evaluation was performed using recent global modeled datasets on wet N deposition rates^9^, while the evaluation of dry deposition was not performed due to a lack of data^30,31^. The evaluation of wet deposition against station measurement includes North America, Europe, Africa, Asia, South America, and forest areas. The major difference in the ACCMIP and Wang *et al*. lies in the emission data used to model the deposition, that leads to differences in the N deposition estimates.

Between the two cropland maps, HYDE is created using simple time-varying land allocation algorithms. It develops land use maps (e.g., cropland) from 10,000 BCE to present. The HYDE dataset is developed for each 100-year from 800 to 1700, at a decadal scale between 1700 and 2000, and at an annual scale from 2000 to 2015. The key input data include historical national level “arable land and permanent crops” and “permanent meadows and pastures” from the Food and Agriculture Organization of the United Nations (FAO)^32^. Additionally, subnational datasets are utilized. These coarser statistics on land use are then converted to finer resolution croplands using European Space Agency (ESA) Land Cover Consortium maps^33,34^ and following a sequence of allocation steps. The ESA land cover provides a land mask for HYDE and is available for three years, but the most recent epoch is used to develop the dataset. For more details on steps, see Goldewijk *et al*.^22^. At a national scale, HYDE dataset has shown consistent estimates with FAO’s land use data but suffers from larger differences compared to other satellite-based products^35^.

The input dataset in LUH2 land-use states includes the HYDE database to develop a historic dataset. The data from HYDE are on a decadal scale from 1700 to 2000 and annually from 2000 to the present. This data was linearly interpolated to establish an annual time series of gridded cell area fractions of different land use types (e.g., cropland). The cropland area fraction includes five different functional types: C3 annuals, C4 annuals, C3 perennials, C4 perennials, and C3 nitrogen fixers. This dataset also takes crop rotation and agricultural management practices into account. Although LUH2 is an improvement over HYDE, it also underestimates cropland area in some regions compared to other studies^36^.

The goal for comparison against site-based observations is to see whether our national N deposition estimates fall in the range of the site-based observations in cropland. Although we don’t anticipate our national estimates to exactly match the values and trends of the measurements from these cropland sites, we do expect them to align within the observed range (i.e., have a similar magnitude and/or exhibit comparable overall trends). Since our study focuses on cropland, we limited our comparison to the sites that are located either in croplands or agricultural lands. Such selection criteria reduce the number of available measurement stations. Additionally, the majority of sites provide wet deposition estimates, and dry deposition measures are not available in the station network sites due to a lack of data^20,30,31^. Despite this fact, we included NADP, ECN, and China’s N wet deposition for comparison. Except for a few sites in EANET that are located in rural areas with both dry and wet deposition^37^; rest of the sites have data for wet precipitation chemistry. These limitations indicate that the measurement of N deposition on agricultural land requires both better spatial coverage worldwide and greater standardization of protocols and data. Hence, for comparison on agricultural/crop land, we have collected and compared our estimates with long-term data on N deposition from the USA, China, the UK, and East Asian sites.

### Methods for estimating N deposition

**Fig. 2:**
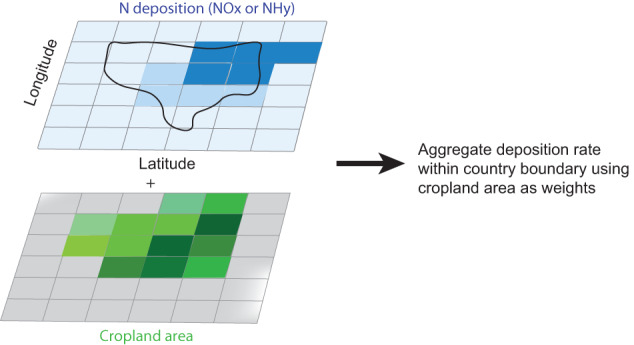
Schematic of aggregating N deposition on cropland.

Following previous studies, the N deposition is estimated by overlaying the N deposition maps, cropland distribution maps, and country boundaries (Fig. 2). Then, using cropland area as weights within a country’s boundary, we aggregate N deposition to a national scale. The N deposition data from Wang *et al*. are available as bulk estimates, whereas the ACCMIP dataset provides deposition in separate forms (i.e., NOx, and NHy). For separate cases, the N deposition is calculated for different forms of reactive N, and then aggregated together to represent bulk N deposition.

### Adjustments to cropland maps

HYDE cropland maps are available at decadal scale from 1960 to 2000, and at annual scale after 2000, until 2017 (Table 2). To create a continuous time series of N deposition, the following adjustments were done to the cropland map:

- 1961–1965: used 1960’s cropland area
- 1966–1975: used 1970’s cropland area
- 1976–1985: used 1980’s cropland area
- 1986–1995: used 1990’s cropland area
- 1996–2000: used 2000’s cropland area
- 2001–2017: used annual cropland maps
- 2018–2020: used 2017 cropland map

The LUH2 cropland map is reported as the fraction of land in a grid cell. These fractions are available for 12 possible land-use categories, including the classification of primary and secondary natural vegetation into forest and non-forest sub-types, pasture into managed pasture and rangeland, and cropland into multiple crop functional types. The fractions falling under the category of cropland (i.e., C3 annual crops, C3 perennial crops, C4 annual crops, C4 perennial crops, C3 nitrogen-fixing crops) were chosen, and the maximum fraction among these five sub-categories was selected to represent cropland. The fraction was converted to land area by multiplying the fraction of cropland in a grid cell by the grid cell’s spatial resolution. LUH2 maps are available at annual scale until 2015. As a result, the cropland maps between 2015 and 2020 are assumed to be the same as in 2015.

### Extrapolation and interpolation of N deposition maps

The data from Wang *et al*. are at decadal scale until 1990, followed by annual scale from 1997 to 2013. To prepare a complete time series by filling in the gaps, we took the following steps:

1. Between 1960–1970, the data for N emission and N deposition maps are unavailable. The N deposition maps are available for two years, 1960 and 1970. Using these two years, the values of N deposition were interpolated between 1961–1969 using Eq. 1.
  1 $${N}_{deposition}={\beta }_{0}+{\beta }_{1}Y$$
  where Y is the year, and *β*_0_ and *β*_1_ are intercept and slope, respectively.
2. Interpolation between 1970–1997: Both the N emission and N deposition maps are available between the years 1970–1997 but for different sets of years. N emissions maps are available annually from 1970 to 1997, while N deposition is only available for four years (i.e., 1970, 1980, 1990 and 1997). Using the common years (i.e., 1970, 1980, 1990 and 1997) considering both the datasets, a relationship between N emission and N deposition was established (Eq. 2). This relationship was used to interpolate values of N deposition for years other than 1970, 1980, 1990, and 1997 using N emission maps that are available annually.
  2 $${N}_{deposition}={\beta }_{0}+{\beta }_{1}{N}_{emission}$$
3. Extrapolation after 2013: Between 1997–2018, both the N emission and N depositions maps are available. However, N deposition maps are available only until 2013. Using the common years (i.e., 1997–2013), a relationship between N emission and N deposition was established (Eq. 2). Then, N deposition for each grid was estimated from 2013 to 2018 using N emission map.
4. For years 2019 and 2020, due to unavailability of N emission data, the N deposition was assumed to be the same as in 2018.

These steps were conducted at gridded level and the deposition rates were aggregated to national scale using the approach mentioned in section *“Methods for estimating N deposition”*.

### Validation of the N deposition products

**Fig. 3:**
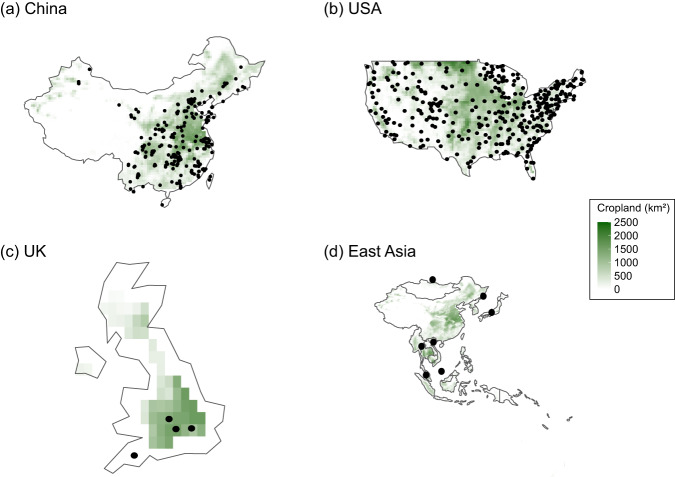
Station network locations in the chosen countries (**a**) China, (**b**) USA, and (**c**) UK, and (**d**) East Asia. The black dots show the location of stations. For reference, a cropland area from LUH2 for year 2000 is added. The gradient from light to dark green shows smaller to larger cropland area.

We used two approaches to validate the N deposition inputs from the four data products. First, we compared the N deposition data products in this study with the site-level observations. Second, we compared the four data products with the N deposition estimated by the other global N budget studies. For the first approach, we collected observation records of N deposition flux over the agricultural lands across China, the UK, the USA, and East Asia including 268, 4, 357, and 7 stations, respectively (Fig. 3 and Table 2). Except the UK, the N deposition flux from the station networks in other regions is expressed similarly to the products in this study. In the United Kingdom, six stations of the Environmental Change Network (ECN: https://ecn.ac.uk) are located on agricultural land (i.e., Drayton, Hillsborough, North Wyke, Porton, Rothamsted, and Wytham). Among them, only Drayton, North Wyke, Rothamsted, and Wytham have precipitation chemistry data available for ammonium, nitrate, and total nitrogen deposition (Fig. 3c). The N deposition data from each station are available on a daily scale in mg L^−1^. To convert the unit to kg N ha^−1^ yr^−1^, the following approach was followed:

1. Total N (kg N ha^−1^ day^−1^) = Total N (mg L^−1^) × Precipitation (*mm*) × 0.01
2. $${\rm{Total}}\;{\rm{N}}\left({\rm{kg}}\;{\rm{N}}\;{{\rm{ha}}}^{-{\rm{1}}}{{\rm{yr}}}^{-{\rm{1}}}\right)=365\times \frac{1}{{\rm{n}}}{\sum }_{1}^{n}{\rm{Total}}\;{\rm{N}}\left({\rm{kg}}\;{\rm{N}}\;{{\rm{ha}}}^{-1}{{\rm{day}}}^{-1}\right)$$

where n is the number of days with available data in a year from all stations. The daily precipitation data were obtained from ECMWF^38^. Each station’s precipitation corresponds to the precipitation value in the grid cell adjacent to the respective station.

For the second approach, we compared the N deposition estimates in this study with the N deposition from 14 global nitrogen budget datasets represented collectively in Zhang *et al*.^14^. The datasets include Zhang Reorganized^13^, Zhang 2015^13^, Lassaletta^39,40^, Lu and Tian^41,42^, Nishina with double cropping^43^, Nishina without double cropping^43^, Conant and Dorich^16^, Bodirsky without forage^11,17^, Bodirsky with forage^11,17^, IMAGE^12^, FAO^44^, Gerber and Mueller^45–47^, Chang *et al*.^48–52^, and NuGIS^53^.

### Sensitivity analysis

The sensitivity analysis examined how N deposition estimates from the four different products affect the assessment of NUE for each country over the past decades. The NUE is defined as the ratio of N removal by the crop (CR) to the sum of N inputs from synthetic fertilizer (SF), manure (MN), biological N fixation (BNF), and atmospheric N deposition (AD) (Eq. 3). We used N fertilizer, N manure, N fixation from FAOSTAT cropland nutrient budget^26^, while varying the N deposition estimates based on the four products developed in this study. Since the FAOSTAT cropland nutrient budget dataset^26^ already includes N deposition from WL, we estimated a reference NUE with WL, and compared this NUE with the NUE estimated by replacing the N deposition with other three products (i.e., AH, AL, and WH) while keeping the remaining three elements (i.e., SF, MN, and BNF) of the N budget the same.

3 $$NUE=\frac{{N}_{CR}}{{N}_{SF}+{N}_{MN}+{N}_{BNF}+{N}_{AD}}$$

With the four sets of NUE (i.e., NUE_WL_, NUE_AH_, NUE_AL_, and NUE_WH_), two parameters were estimated to evaluate the changes in NUE due to differences in N deposition.

1. Difference in NUE = NUE_p ϵ AL, AH, WH_–NUE_WL_
2. Correlation of NUE (NUE_p ϵ AL, AH, WH_, NUE_WL_)

## Data Records

Data are available at Vishwakarma *et al*.^54^, Quantifying nitrogen deposition inputs to cropland: A national scale dataset from 1961 to 2020, Dryad, Dataset, 10.5061/dryad.msbcc2g1x.

The data contain N deposition (kg N ha^−1^ yr^−1^) in cropland. Each file has rows for countries and columns for years. The NaN stands for “Not a Number”.

File description:

1_AH.xlsx: includes the nitrogen deposition (kg N ha^−1^ yr^−1^) data from ACCMIP and HYDE

2_AL.xlsx: includes the nitrogen deposition (kg N ha^−1^ yr^−1^) data from ACCMIP and LUH2

3_WH.xlsx: includes the nitrogen deposition (kg N ha^−1^ yr^−1^) data from Wang *et al*. and HYDE

4_WL.xlsx: includes the nitrogen deposition (kg N ha^−1^ yr^−1^) data from Wang *et al*. and LUH2

## Technical Validation

### Spatiotemporal differences in N deposition

**Fig. 4:**
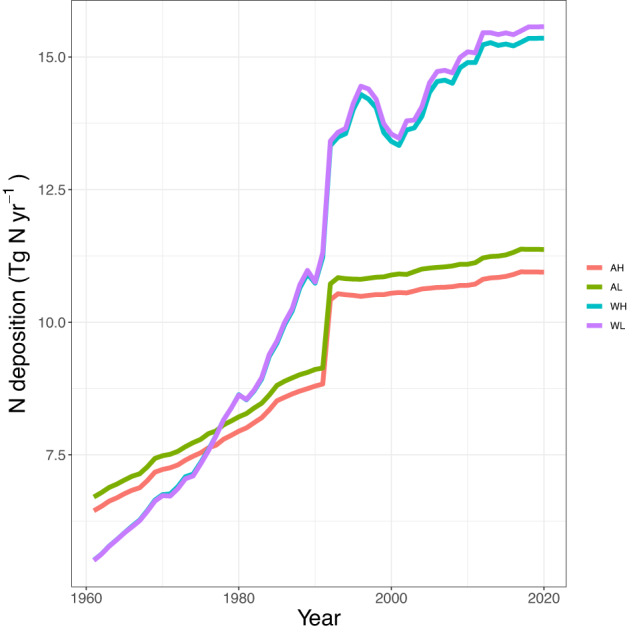
Global N deposition on cropland from 1961 to 2020. The acronyms in the legend are AH: ACCMIP + HYDE, AL: ACCMIP + LUH2, WH: Wang *et al*. + HYDE, and WL: Wang *et al*. + LUH2.

The four products, namely **AH** (ACCMIP + HYDE), **AL** (ACCMIP + LUH2), **WH** (Wang *et al*. + HYDE), **WL** (Wang *et al*. + LUH2), show substantial differences in global N deposition estimates in cropland (Fig. 4). For example, the difference in global N deposition between ACCMIP and Wang *et al*. in the year 2000 was approximately 3 Tg N yr^−1^, which increased over time, particularly after 1992. These variations are primarily caused by the differences in simulation and modeling approaches to develop N deposition maps using emission inventories. Regardless of which deposition maps were used, the N deposition estimates with the HYDE map as weights were consistently lower than compared to using the LUH2 map. However, the differences between the two maps derived from Wang *et al*. deposition estimates (i.e., WH vs. WL) are small.

**Fig. 5:**
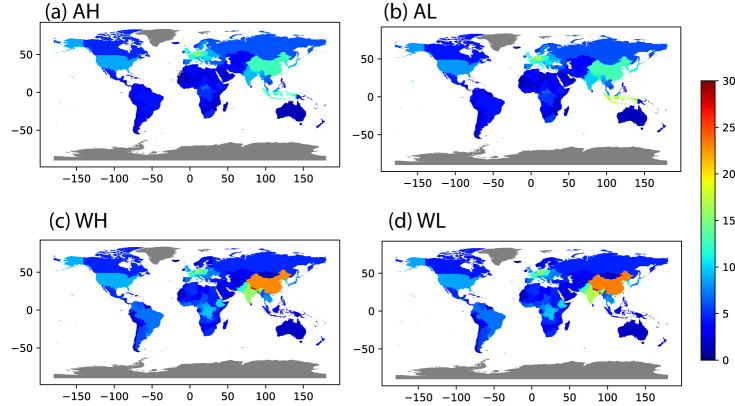
Cropland N deposition (kg N ha^–1^ yr^–1^) at country scale for the year 2000 using (**a**) AH: ACCMIP + HYDE, (**b**) AL: ACCMIP + LUH2, (**c**) WH: Wang *et al*. + HYDE, and (**d**) WL: Wang *et al*. + LUH2.

When comparing N deposition for a specific year, these differences on a global scale are also visible spatially. Figure 5a,b shows an example of estimates for the year 2000 using ACCMIP deposition data in combination with two cropland maps (i.e., HYDE and LUH2) on the national scale. The estimates for most countries appear to be the same, apart from Indonesia, which is higher with LUH2 instead of HYDE. Similar to ACCMIP, N deposition estimates at country scale obtained from Wang *et al*. are shown in Fig. 5c,d. Irrespective of which cropland map is used, the estimates from Wang *et al*. are similar. When Wang *et al*. products are compared to ACCMIP products, major differences are mostly found in Asian countries (e.g., China, India, and Pakistan), where Wang *et al*. estimates are higher than ACCMIP estimates.

### Evaluation of the N deposition products

**Fig. 6:**
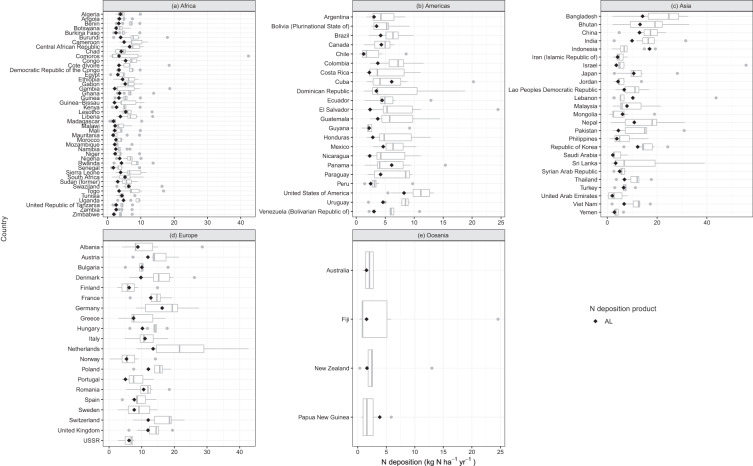
Comparison of the product AL (i.e., ACCMIP + LUH2) and other studies’ total N deposition (Table 1) for year 2000. The black diamond shows product AL. Each sub-plot indicates countries in different regions: (**a**) Africa, (**b**) Americas, (**c**) Asia, (**d**) Europe, and (**e**) Oceania.

**Fig. 7:**
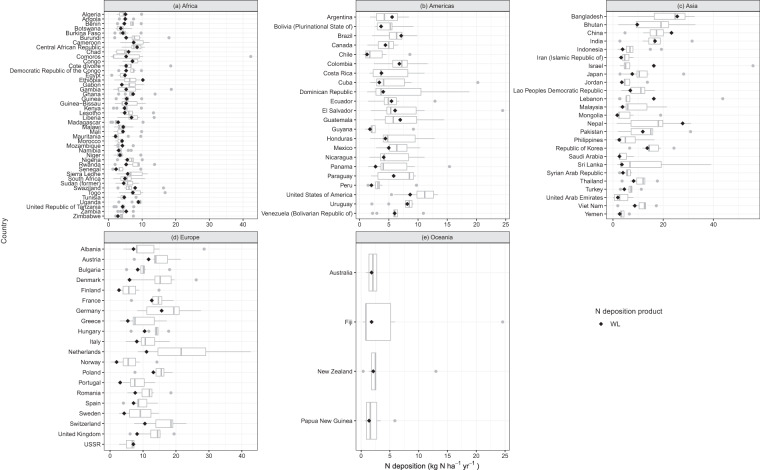
Comparison of the product WL (Wang *et al*. + LUH2) and other studies’ total N deposition (Table 1) for year 2000. The black diamond shows product WL. Each sub-plot indicates countries in different regions: (**a**) Africa, (**b**) Americas, (**c**) Asia, (**d**) Europe, and (**e**) Oceania.

Large variation exists in the national N deposition estimates from different products. Such uncertainty stems primarily from the various approaches and assumptions used to estimate N deposition. The AL product is at the lower end of the distribution of N deposition from 14 previously developed estimates, whereas WL is either around the median or on the higher side (Figs. 6 and 7). For most regions, the WL is in the interquartile range of 14 other studies. Europe’s estimates are outside the interquartile range and on the lower end. However, with AL, the estimates for Europe are slightly higher and within the interquartile range. In the remaining regions, AL is almost always lower than the 14 other nitrogen deposition estimates. Overall, WL estimates compare favorably to the remaining 14 N budget datasets with N deposition.

### Comparison with station networks

All four data products either slightly overestimated or were within the range of station records of N deposition. However, for most practical purposes these differences are probably acceptable.

**Fig. 8:**
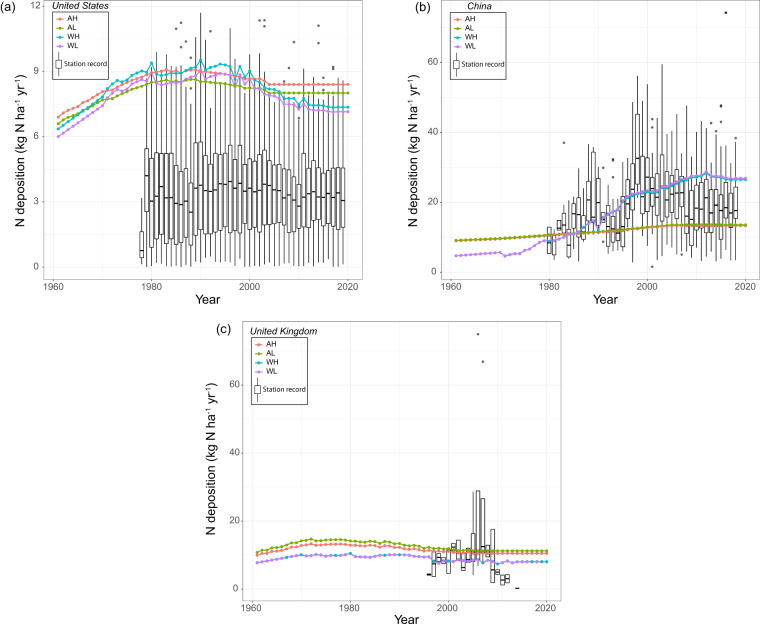
Comparison of station measurements with the N deposition maps aggregation for (**a**) United States, (**b**) China, and (**c**) United Kingdom. Each colored dot represents estimate for each year from different deposition and cropland maps. Boxplot represents distribution of N deposition from stations considered for comparison. These boxplots only represent wet N deposition due to unavailability of dry deposition from these networks; hence, for some years the ground station values are systematically lower than our product estimates. The acronyms in legends are AH: ACCMIP + HYDE; AL: ACCMIP + LUH2; WH: Wang *et al*. + HYDE; and WL: Wang *et al*. + LUH2.

In the USA, all four products overestimated N deposition from the station network (Fig. 8a). In fact, until 2002, the values fall outside the upper bounds of the station records. The four products appear to be within the range for years 2003, 2011, 2013, and 2018. They are, however, above the 75^th^ percentile. Hence, a clear indication of overestimation is seen in the USA, but, for most application purposes, the products’ N deposition estimates are only few kg N per ha cropland higher than station records.

In China, products utilizing the ACCMIP-based deposition map were not only substantially lower than the Wang *et al*.-based products, but they were also at the lower end of the distribution of the station records. The products based on Wang *et al*. were comparable to station data estimates until 2008 but appear to be higher than the 75^th^ percentile after 2008 (Fig. 8b).

In the UK, the N deposition products from both ACCMIP and Wang *et al*. fell within the range of the observed N deposition station records prior to 2009 and were approximately 5 kg N ha^−1^ higher than the median of the observed values after 2010 (Fig. 8c). In the observation data, a few cases have exceptionally high values of N deposition, which is due to the higher precipitation on some days in a year resulting in higher N deposition.

**Fig. 9:**
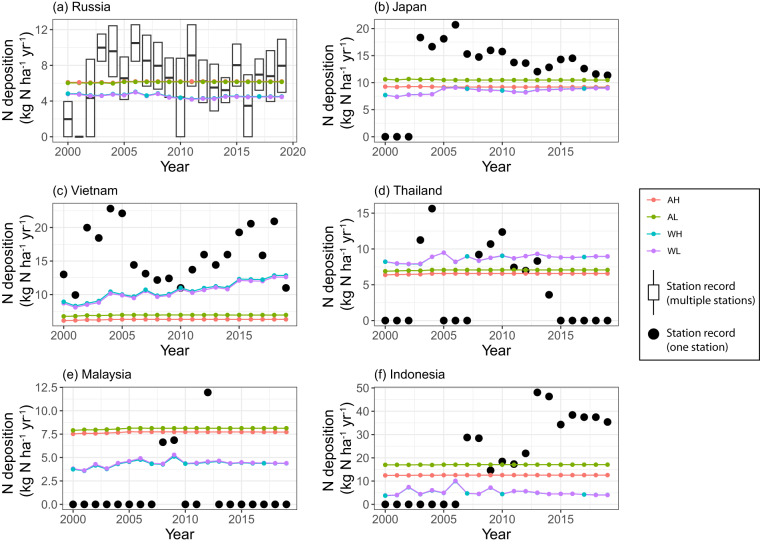
Comparison of station measurement from EANET station-network with the N deposition maps aggregation. Each colored dot represents estimate for each year from different deposition and cropland maps. The black dot here indicates data from station network. Boxplot represents distribution of N deposition (dry + wet) from stations considered for comparison. The acronyms in legends are AH: ACCMIP + HYDE; AL: ACCMIP + LUH2; WH: Wang *et al*. + HYDE; and WL: Wang *et al*. + LUH2. Note: Except for Russia, which has two sites in rural area, the remaining countries have only one site in rural area.  .

For countries in the EANET ground station network, there is either one or two stations data available in the rural sites to evaluate the N deposition on agricultural crops^37^. In these countries, the N deposition products from Wang *et al*. are either lower or similar to ACCMIP estimates except in Vietnam and Thailand (Fig. 9). This indicates underestimation of N deposition in some of the Southeast Asian countries from the Wang *et al*. products. Moreover, none of the estimates from both ACCMIP and Wang *et al*. are outside the range of N deposition from the sites. This shows a reasonable values of N deposition from the products developed in this study.

Overall, the total N deposition (dry + wet) from four products in China and the UK are close to the median wet deposition estimates from station networks (Table 3). However, we did not find a close match in the USA. The main reason for the systematically lower ground station values in the USA is that they do not include dry deposition. Although the values of our products in China fall close to the median, after 2010, the bulk N deposition estimates in China (Fig. 8b) from Wang *et al*.-based products are rising above the median of the deposition record from station network. This could be because of recent rising emissions in China and its direct correlation with higher deposition rates^55^. Hence, we think if dry depositions are also accounted for in the sites, the deposition from four products might match the site values. Furthermore, the UK’s emissions (rainfall rate and frequency) are lower (higher) than the USA and China^27^. That’s why, the deposition values from our products are matching with the wet deposition in the UK.

**Table 3:** Comparison of measured and estimated annual N deposition (kg N ha^–1^ yr^–1^) for year 2000.

Country	AH	AL	WH	WL	Station network (median)
China	12.71	12.95	22.93	23.29	27.18
USA	8.67	8.24	9.23	8.64	3.50
UK	11.09	11.91	7.61	8.19	8.05

Note: EANET is not compared here because of single station value available in each country.

### Sensitivity analysis

**Fig. 10:**
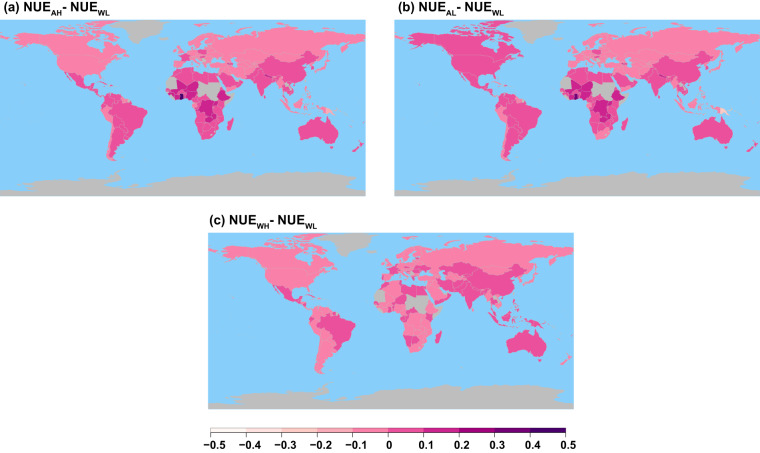
Map of difference in NUE (i.e., NUE_p ϵ AL, AH, WH_-NUE_WL_) for year 2000 in absolute terms. The acronyms in the plot are AH: ACCMIP + HYDE; AL: ACCMIP + LUH2; WH: Wang *et al*. + HYDE; and WL: Wang *et al*. + LUH2.

The differences in NUE are higher with the N deposition map from ACCMIP (Fig. 10). When using either HYDE or LUH2 cropland map as weights for aggregation of ACCMIP N deposition maps, the difference in NUE is closer to zero for most of the countries except for a few countries in Africa and Eurasia. In contrast, the NUE difference between WH and WL products is in the range of 0 and 0.1 in absolute terms. Considering the impact of N deposition maps, both the ACCMIP and Wang *et al*. have smaller impact on estimates of  NUE for the majority of countries except for few countries in Africa and Eastern Europe. For example, in year 2000, difference in cropland NUE for China is 0.19% when Wang *et al*. + HYDE deposition is used, while the difference is 1% when ACCMIP + HYDE is used.

**Fig. 11:**
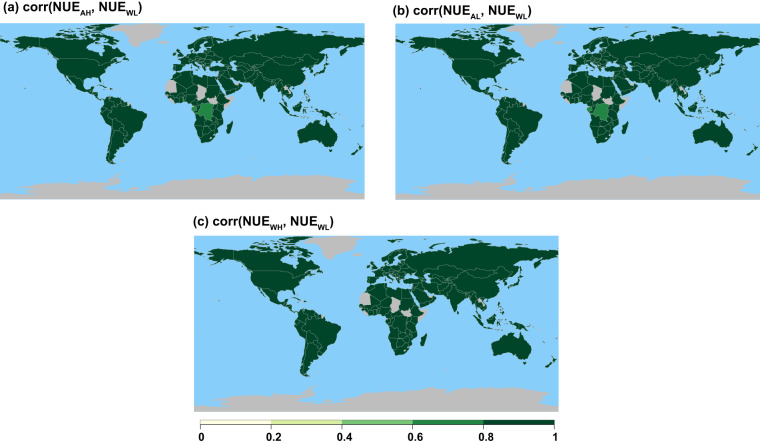
Map of correlation of NUE. Each figure shows correlation between NUE_p ϵ AH, AL, WH_ and NUE_WL_, where NUE_p ϵ AH, AL, WH_ is estimated using these N deposition products: AH: ACCMIP + HYDE, AL: ACCMIP + LUH2, and WH: Wang *et al*. + HYDE, respectively.

Similar to differences in NUE, the correlation between NUE_WL_ and NUE estimated by using the other three products did not vary a lot. Despite replacement of N deposition from AH, AL, and WH in the NUE estimation, all products show high correlation (r > 0.8) in the majority of countries when the reference NUE (i.e., NUE _WL_) for the period of 1961–2020 obtained from FAOSTAT’s cropland nutrient budget dataset^26^ were correlated with the other three N deposition products (Section *“Sensitivity Analysis”*, and Fig. 11). This indicates less sensitivity of NUE and the general usability of any of these products for global scale NUE assessment.

## Usage Notes

Within this study, we developed four N deposition products at national scale; **AH**: ACCMIP + HYDE, **AL**: ACCMIP + LUH2, **WH**: Wang *et al*. + HYDE, and **WL**: Wang *et al*. + LUH2. We recommend using the **WL** product for most general studies of global and regional cropland N budgets and NUE. It has already been adopted in the Cropland Nutrient Budget database jointly released^26^ by FAO and IFA in 2022, for the following reasons: (1) the Wang *et al*. estimates are closer to the remaining existing datasets for the majority of regions, particularly Asian countries, whereas the estimates obtained using ACCMIP are on the lower side of the distribution of N deposition compared to other datasets, with minimal change after 2005, (2) the LUH2 cropland map is available at an annual time scale, which serves the purpose of this study, while the HYDE map is only available at a decadal scale until 2000, and annually from 2000–2017, and (3) the WL product was within the range of ground estimates of N deposition from the station networks in multiple countries (i.e., China, the UK, the USA, and East Asia). Overall, however, the sensitivity analysis showed small impacts of the four N deposition products on estimating NUE at national to global scales.

The raw N deposition maps used in this study are derived from N emissions. Emissions may originate from a variety of sources, including transportation, wastewater handling, direct soil emissions etc.^27^. Our country-specific deposition estimates, which rely on the rate of emissions, may have overlap effects (i.e., N deposition in a country could include re-deposition of N emission either within a country boundary or from other neighboring countries) because these emissions are not border-restricted. Hence, the four products in this work do not pertain an equal relationship to national emission rates, which could be lower or higher.

Admittedly, the existing N deposition data products are still limited in providing more accurate global scale estimates. Improving them will require better field-scale measurements techniques^9^ and networks, modeling improvements^10^, and updated cropland maps^56^.

## Data Availability

Codes can be accessed at https://github.com/svish91/UMCES_IFA_2021_Ndep.

## References

[1] Zhang X (2020). Quantifying Nutrient Budgets for Sustainable Nutrient Management. Global Biogeochem Cycles.

[2] Galloway, J. N., Leach, A. M., Bleeker, A. & Erisman, J. W. A chronology of human understanding of the nitrogen cycle. *Philosophical Transactions of the Royal Society B: Biological Sciences* **368** (2013). 10.1098/rstb.2013.0120 PMC3682740 23713118

[3] Li Y (2016). Increasing importance of deposition of reduced nitrogen in the United States. Proc Natl Acad Sci USA.

[4] Banger K (2015). Magnitude, Spatiotemporal Patterns, and Controls for Soil Organic Carbon Stocks in India during 1901–2010. Soil Science Society of America Journal.

[5] Liu, L. *et al*. Exploring global changes in agricultural ammonia emissions and their contribution to nitrogen deposition since 1980. *Proceedings of the National Academy of Sciences* **119** (2022). 10.1073/pnas.2121998119 PMC9169101 35344440

[6] Yadav MR (2017). Strategies for improving nitrogen use efficiency: A review. Agricultural Reviews.

[7] United Nations. *Report of the United Nations Environment Assembly of the United Nations Environment Programme*. https://wedocs.unep.org/bitstream/handle/20.500.11822/40673/N2233507.pdf?sequence=1&isAllowed=y (2022).

[8] United Nations. COP15 ends with landmark biodiversity agreement. https://www.unep.org/news-and-stories/story/cop15-ends-landmark-biodiversity-agreement (2022).

[9] Vet R (2014). A global assessment of precipitation chemistry and deposition of sulfur, nitrogen, sea salt, base cations, organic acids, acidity and pH, and phosphorus. Atmos Environ.

[10] Lamarque JF (2013). Multi-model mean nitrogen and sulfur deposition from the atmospheric chemistry and climate model intercomparison project (ACCMIP): Evaluation of historical and projected future changes. Atmos Chem Phys.

[11] Bodirsky B (2012). N2O emissions from the global agricultural nitrogen cycle-current state and future scenarios. Biogeosciences.

[12] Bouwman L (2013). Exploring global changes in nitrogen and phosphorus cycles in agriculture induced by livestock production over the 1900–2050 period. Proceedings of the National Academy of Sciences.

[13] Zhang X (2015). Managing nitrogen for sustainable development. Nature.

[14] Zhang X (2021). Quantification of global and national nitrogen budgets for crop production. Nat Food.

[15] Zhang Q (2021). Atmospheric nitrogen deposition: A review of quantification methods and its spatial pattern derived from the global monitoring networks. Ecotoxicol Environ Saf.

[16] Conant RT, Berdanier AB, Grace PR (2013). Patterns and trends in nitrogen use and nitrogen recovery efficiency in world agriculture. Global Biogeochem Cycles.

[17] Bodirsky, B. L. *et al*. Reactive nitrogen requirements to feed the world in 2050 and potential to mitigate nitrogen pollution. *Nat Commun* **5** (2014). 10.1038/ncomms4858 24819889

[18] Wang Q (2020). Data-driven estimates of global nitrous oxide emissions from croplands. Natl Sci Rev.

[19] Shang Z (2019). Weakened growth of cropland-N2O emissions in China associated with nationwide policy interventions. Glob Chang Biol.

[20] Wang R (2017). Global forest carbon uptake due to nitrogen and phosphorus deposition from 1850 to 2100. Glob Chang Biol.

[21] Hurtt, G. C. *et al*. *Harmonization of global land use change and management for the period 850-2100 (LUH2) for CMIP6*. *Geoscientific Model Development***vol. 13** (2020).

[22] Goldewijk KK, Beusen A, Doelman J, Stehfest E (2017). Anthropogenic land use estimates for the Holocene - HYDE 3.2. Earth Syst Sci Data.

[23] de Vries W, Schulte-Uebbing L, Kros H, Voogd JC, Louwagie G (2021). Spatially explicit boundaries for agricultural nitrogen inputs in the European Union to meet air and water quality targets. Science of The Total Environment.

[24] Lu C (2012). Effect of nitrogen deposition on China’s terrestrial carbon uptake in the context of multifactor environmental changes. Ecological Applications.

[25] Sheeder SA, Lynch JA, Grimm J (2002). Modeling Atmospheric Nitrogen Deposition and Transport in the Chesapeake Bay Watershed. J Environ Qual.

[26] FAO (Food and Agriculture Organization). FAOSTAT: Cropland nutrient budget. In: FAO. Rome. https://www.fao.org/faostat/en/#data/ESB (2022).

[27] EDGAR. *Global Air Pollutant Emissions. EDGAR - Emissions Database for Global Atmospheric Research. EDGAR v6.1.* https://edgar.jrc.ec.europa.eu/index.php/dataset_ap61 (2020).

[28] Hourdin F (2006). The LMDZ4 general circulation model: climate performance and sensitivity to parametrized physics with emphasis on tropical convection. Clim Dyn.

[29] Hauglustaine DA, Balkanski Y, Schulz M (2014). A global model simulation of present and future nitrate aerosols and their direct radiative forcing of climate. Atmos Chem Phys.

[30] Dentener, F. *et al*. Nitrogen and sulfur deposition on regional and global scales: A multimodel evaluation. *Global Biogeochem Cycles* **20** (2006).

[31] Lamarque J-F (2005). Assessing future nitrogen deposition and carbon cycle feedback using a multimodel approach: Analysis of nitrogen deposition. J Geophys Res.

[32] FAOSTAT. FAOSTAT: Statistical database. *FAOSTAT: Statistical database*. (2018).

[33] European Space Agency. ESA: Three global LC maps for the 2000, 2005 and 2010 epochs. https://www.esa-landcover-cci.org/?q=node/158 (2016).

[34] Hollmann R (2013). The ESA Climate Change Initiative: Satellite Data Records for Essential Climate Variables. Bull Am Meteorol Soc.

[35] Li S, He F, Zhang X, Zhou T (2019). Evaluation of global historical land use scenarios based on regional datasets on the Qinghai–Tibet Area. Science of The Total Environment.

[36] Qiu Y, Feng J, Yan Z, Wang J (2023). Assessing the land-use harmonization (LUH) 2 dataset in Central Asia for regional climate model projection. Environmental Research Letters.

[37] Jin-soo, P. *et al*. *The Fourth Periodic Report on the State of Acid Deposition in East Asia Part I: Regional Assessment*. (2021).

[38] ECMWF. ERA-Interim. *ERA-Interim Reanalysis Project* https://www.ecmwf.int/en/forecasts/datasets/reanalysis-datasets/era-interim (2019).

[39] Lassaletta L (2016). Nitrogen use in the global food system: past trends and future trajectories of agronomic performance, pollution, trade, and dietary demand. Environmental Research Letters.

[40] Lassaletta, L., Billen, G., Grizzetti, B., Anglade, J. & Garnier, J. 50 year trends in nitrogen use efficiency of world cropping systems: The relationship between yield and nitrogen input to cropland. *Environmental Research Letters* **9** (2014).

[41] Lu C, Tian H (2017). Global nitrogen and phosphorus fertilizer use for agriculture production in the past half century: Shifted hot spots and nutrient imbalance. Earth Syst Sci Data.

[42] Zhang B (2017). Global manure nitrogen production and application in cropland during 1860–2014: a 5 arcmin gridded global dataset for Earth system modeling. Earth Syst Sci Data.

[43] Nishina K, Ito A, Hanasaki N, Hayashi S (2017). Reconstruction of spatially detailed global map of NH4+ and NO3− application in synthetic nitrogen fertilizer. Earth Syst Sci Data.

[44] FAOSTAT. *Soil nutrient budget: Global, regional and country trends, 1961–2018*. https://www.fao.org/3/cb4475en/cb4475en.pdf (2021).

[45] Mueller, N. D. *et al*. A tradeoff frontier for global nitrogen use and cereal production. *Environmental Research Letters* **9** (2014).

[46] Mueller ND (2012). Closing yield gaps through nutrient and water management. Nature.

[47] West PC (2014). Leverage points for improving global food security and the environment. Science (1979).

[48] Skalský, R. *et al*. *Geo-bene global database for bio-physical modeling v. 1.0. Concepts, methodologies and data. The GEO-BENE database report*. https://geo-bene.project-archive.iiasa.ac.at/files/Deliverables/Geo-BeneGlbDb10 (DataDescription).pdf (2008).

[49] Herrero M (2013). Biomass use, production, feed efficiencies, and greenhouse gas emissions from global livestock systems. Proceedings of the National Academy of Sciences.

[50] Valin H (2013). Agricultural productivity and greenhouse gas emissions: trade-offs or synergies between mitigation and food security?. Environmental Research Letters.

[51] Havlík P (2014). Climate change mitigation through livestock system transitions. Proceedings of the National Academy of Sciences.

[52] Chang J (2021). Reconciling regional nitrogen boundaries with global food security. Nat Food.

[53] International Plant Nutrition Institute (IPNI). A nutrient use information system (NuGIS) for the U.S. http://www.ipni.net/nugis (2012).

[54] Vishwakarma S, Zhang X, Dobermann A, Heffer P, Zhou F (2022). Dryad.

[55] Granier C (2011). Evolution of anthropogenic and biomass burning emissions of air pollutants at global and regional scales during the 1980–2010 period. Clim Change.

[56] Tubiello FN (2023). Measuring the world’s cropland area. Nat Food.

[57] Ramankutty N, Evan AT, Monfreda C, Foley JA (2008). Farming the planet: 1. Geographic distribution of global agricultural lands in the year 2000. Global Biogeochem Cycles.

[58] Bouwman, A. F., Beusen, A. H. W. W. & Billen, G. Human alteration of the global nitrogen and phosphorus soil balances for the period 1970–2050. *Global Biogeochem Cycles* **23** (2009).

[59] Klein Goldewijk K, Beusen A, van Drecht G, de Vos M (2011). The HYDE 3.1 spatially explicit database of human-induced global land-use change over the past 12,000 years. Global Ecology and Biogeography.

[60] Portmann FT, Siebert S, Döll P (2010). MIRCA2000-Global monthly irrigated and rainfed crop areas around the year 2000: A new high-resolution data set for agricultural and hydrological modeling. Global Biogeochem Cycles.

[61] Monfreda C, Ramankutty N, Foley JA (2008). Farming the planet: 2. Geographic distribution of crop areas, yields, physiological types, and net primary production in the year 2000. Global Biogeochem Cycles.

[62] Eyring V (2013). Overview of IGAC/SPARC Chemistry-Climate Model Initiative (CCMI) Community Simulations in Support of Upcoming Ozone and Climate Assessments. SPARC newsletter.

[63] Hurtt GC (2011). Harmonization of land-use scenarios for the period 1500–2100: 600 years of global gridded annual land-use transitions, wood harvest, and resulting secondary lands. Clim Change.

[64] Siebert S, Portmann FT, Döll P (2010). Global Patterns of Cropland Use Intensity. Remote Sens (Basel).

[65] Rennie S (2017). NERC Environmental Information Data Centre. (Dataset).

[66] NADP. *National Trends Network: A long-term record of precipitation chemistry across the United States.* https://nadp.slh.wisc.edu/networks/national-trends-network/ (2021).

[67] Wen, Z. *et al*. Changes of nitrogen deposition in China from 1980 to 2018. *Environ Int* **144** (2020). 10.1016/j.envint.2020.106022 32795750

